# Opening the treasure chest: A DNA-barcoding primer set for most higher taxa of Central European birds and mammals from museum collections

**DOI:** 10.1371/journal.pone.0174449

**Published:** 2017-03-30

**Authors:** Sylvia Schäffer, Frank E. Zachos, Stephan Koblmüller

**Affiliations:** 1 Institute of Zoology, University of Graz, Universitätsplatz 2, Graz, Austria; 2 Natural History Museum Vienna, Burgring 7, Vienna, Austria; Chang Gung University, TAIWAN

## Abstract

DNA-barcoding is a rapidly developing method for efficiently identifying samples to species level by means of short standard DNA sequences. However, reliable species assignment requires the availability of a comprehensive DNA barcode reference library, and hence numerous initiatives aim at generating such barcode databases for particular taxa or geographic regions. Historical museum collections represent a potentially invaluable source for the DNA-barcoding of many taxa. This is particularly true for birds and mammals, for which collecting fresh (voucher) material is often very difficult to (nearly) impossible due to the special animal welfare and conservation regulations that apply to vertebrates in general, and birds and mammals in particular. Moreover, even great efforts might not guarantee sufficiently complete sampling of fresh material in a short period of time. DNA extracted from historical samples is usually degraded, such that only short fragments can be amplified, rendering the recovery of the barcoding region as a single fragment impossible. Here, we present a new set of primers that allows the efficient amplification and sequencing of the entire barcoding region in most higher taxa of Central European birds and mammals in six overlapping fragments, thus greatly increasing the value of historical museum collections for generating DNA barcode reference libraries. Applying our new primer set in recently established NGS protocols promises to further increase the efficiency of barcoding old bird and mammal specimens.

## Introduction

DNA-barcoding, the sequencing of standardized, species-specific parts of the genome [1], facilitates the rapid and cost-effective genetic characterization of biodiversity, with a wide spectrum of potential applications in biodiversity research, conservation, biosecurity and applied sciences (e.g., [2–7]). Since the introduction of the concept of DNA-barcoding in 2003 [1], huge efforts have been directed towards building a DNA barcode reference library for all eukaryotes based on well-identified specimens [8], which can then be used for identifying unknown specimens at the species level or assign sequences obtained from metabarcoding approaches to taxa [9]. What sets barcoding databases apart from other databases that store genetic data is that in addition to species name and genetic information (i.e. the barcode), a variety of other information and, most importantly, a link to the voucher specimen deposited in a natural history collection is obligatorily provided [8].

Hence, historical museum collections, accumulated over decades to centuries and expertly identified and curated, represent an extremely valuable source of tissue for molecular studies in general [10] and DNA-barcoding activities in particular [11], not least because sampling a sufficient number of specimens for DNA-barcoding studies might be very time-consuming and costly, and even great efforts may not guarantee sufficiently complete sampling in a short period of time. Furthermore, in some cases, collecting fresh (voucher) material from vertebrates, and birds and mammals in particular, is difficult to (nearly) impossible because of current animal welfare and conservation regulations and the fact that certain species are so rare that collecting them for barcoding studies is not advisable from a conservation point of view. Moreover, the scientific value of barcode databases would be greatly enhanced if species were also represented by sequences of the respective type material, especially the holotype [12], as this might facilitate the correct application of taxon names in problematic cases (e.g., [13–15]).

Despite the widely acknowledged value of historical museum collections for DNA-barcoding projects, most DNA-barcoding studies published to date have mainly relied on fresh material obtained via extensive fieldwork, because DNA in museum specimens tends to degrade within a few years, resulting in often limited DNA quantity and quality. Both natural postmortem processes and customary preservation methods cause degradation of DNA [16–18], such that recovering sequence information from historical museum specimens turned out to be a laborious and time-consuming task, if possible at all [19]. The ability to recover DNA sequences from historical material in part depends on the size of the PCR product targeted. Thus, smaller amplicon sizes usually imply a greater amplification success. This is also true for the barcoding region. Amplification of the entire barcoding region is often impossible in museum samples, such that several short (typically <200 bp) overlapping fragments have to be amplified and sequenced, and assembled into a barcode (e.g., [20–22]), thus multiplying the effort per sample necessary to generate a DNA barcode from old museum material. Furthermore, with a few exceptions (e.g., [20,22]), (semi-)universal primers for amplifying and sequencing these short fragments are largely lacking for most taxa, further preventing the large-scale use of historical museum material in DNA-barcoding studies.

A ~650 bp region at the 5’ end of the mitochondrial cytochrome c oxidase subunit 1 gene (COI) is commonly used as the DNA-barcoding region for most animal taxa, including vertebrates [1]. Here, we present the development of PCR primer sets that reliably amplify the barcoding region in most Central European birds and mammals in six overlapping fragments and, therefore, greatly extend the utility of historical bird and mammal specimens from museum collections for large-scale barcoding studies.

## Material and methods

### Ethics statement

Apart from dead animals collected under a permit issued by the provincial government of Styria, only catalogued museum samples or pieces of meat (cattle and wild boar, both from Austria) bought at a supermarket (Interspar, Wienerstrasse, Graz, Austria) were used in this study. Therefore, no further permits were needed.

### Primer design

**Fig 1:**
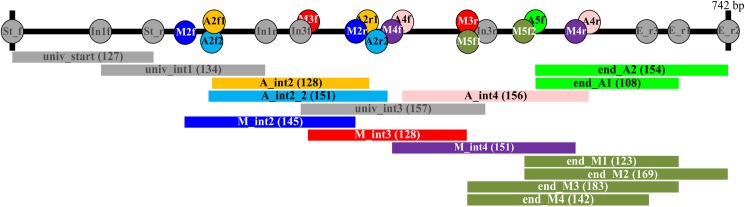
Relative position of the fragments used to amplify the barcoding region in birds and mammals in six overlapping fragments. Fragment length (in bp) without primers is given in parentheses.

New primer sets for amplifying the mitochondrial cytochrome c oxidase I (COI) barcoding region in Central European birds and mammals in six overlapping fragments were designed based on alignments of full DNA barcodes of representatives of all bird and mammal orders occurring in Central Europe, downloaded from BOLD (www.boldystems.org) (S1 Table). To get an idea of the overall variability along the barcoding fragment, we conducted a sliding window analysis in DnaSP 5.10 [23]. Therefore, nucleotide diversities were calculated over all downloaded barcode sequences (S1 Table) based on a window of 20 bp which was then moved by 1 bp increments across the whole alignment. Primer design for birds and mammals was conducted separately. We searched for highly conserved regions as potential primer binding sites and aimed at a maximum fragment size of 200 bp. Some bird primers were partially modified from [20]; the rest of the bird primers as well as all mammal primers were newly designed in the present study. Slightly modified versions of the primer pair for the mini-barcodes in [24] were used to amplify the starting region of the COI in both birds and mammals. Potential primers were evaluated and optimized using the program FastPCR [25], which allows for the detection of primer secondary structures, hairpins, self-dimers and cross-dimers in primer pairs, as well as conducting *in-silico* PCRs. All primers were tagged with M13-tails (M13F: 5’-TGTAAAACGACGGCCAGT-3’, M13R: 5’-CAGGAAACAGCTATGAC-3’; [26]) to allow for efficient high-throughput sequencing. For details on all primers see Fig 1 and Table 1.

**Table 1:** Primers used to amplify the barcoding region in Central European birds and mammals in six overlapping fragments (see Fig 1).

Fragment	fwd	Sequence 5´to 3´ (base pair positions within the 742 bp COI fragment)	rev	Sequence 5´to 3´ (base pair positions within the 742 bp COI fragment)	Reference	Ta
univ_start	St_f	CYNCWAMCCACAARGAYATNGGNAC (-24-0)	St_r	GAARATYATNAYGAANGCRTGNGC (128–151)	Meusnier et al. 2008*	48°C/50°C
univ_int1	In1f	GGNGAYGAYCARATNTACAATGT (95–117)	In1r	GGNGGNAGNAGTCARAARC (252–270)	this study	48°C/50°C
A_int2	A2f1	CCNGACATRGCNTTCCCNCG (218–237)	A2r1	GCNARGTCNACNGANGCNCCNG (366–387)	Patel et al. 2010*	46°C/48°C
A_int2_2	A2f2	GNGCNCCNGAYATRGCNTTYCC (213–234)	A2r2	CNGCNAGRTGNAGNGARAARATNGC (386–410)	this study	46°C/48°C
M_int2	M2f	GGNAAYTGACTNGTNCCNCT (185–204)	M2r	CNGCRTGNGCNAGRTTNCC (350–368)	this study	46°C/48°C
univ_int3	In3f	GGNGTNGGNACNGGNTGAAC (311–330)	In3r	GATCANACGAANAGNGGNGTYTG (488–510)	Patel et al. 2010*	46°C/48°C
M_int3	M3f	GGNACNGGNTGAACNGTNTACC (317–338)	M3r	GRTATTGNGANATNGCNGGNGG (467–488)	this study	46°C/48°C
A_int4	A4f	CNTCNATCCTNGGNGCAATYAAC (417–439)	A4r	GCNGGGTCRAAGAANGTNGTGTT (596–618)	Patel et al. 2010*	48°C/50°C
M_int4	M4f	GCNGGNGTNTCNTCNATTYTAGG (407–429)	M4r	ARGTTGTRTTYARGTTNCGGTCYGT (581–605)	this study	48°C/50°C
end_A1	A5f	GGNATCACNATRCTNCTNACNGACCG (563–588)	E_r1	GTRKGAGATRATTCCGAAKCC (697–716)	this study	46°C/48°C
end_A2	A5f		E_r2	ATNCCTATGTANCCGAATGGRTCTTT (743–769)	Patel et al. 2010*	46°C/48°C
end_M1	M5f2	CNGTNCTAGCNGCYGGNATYACNAT (549–573)	E_r1		this study	46°C/48°C
end_M2	M5f2		E_r2			46°C/48°C
end_M3	M5f1	CNCARTAYCAAACNCCNCTNTTYGT (488–513)	E_r1		this study	46°C/48°C
end_M4	M5f1		E_r3	TANACNTCNGGNTGNCCNAANAATCA (656–681)	this study	46°C/48°C

fwd, forward primer; rev, reverse primer; primer positions (bp) indicate the position within the COI fragment amplified by primers St_f and E_r2 (total length of this fragment, 742 bp); Ta, annealing temperature

*, new primer is a modified version of a previously published primer.

Degenerate bases are indicated by the appropriate International Union of Pure and Applied Chemistry (IUPAC) single-letter designation: R = A or G; Y = C or T; M = A or C; K = G or T; W = A or T; N = A, C, G, or T

All primers were tagged with M13-tails (fwd, M13F: 5’-TGTAAAACGACGGCCAGT-3’; rev, M13R: 5’-CAGGAAACAGCTATGAC-3’; Messing 1983)

### Specimen acquisition

A total of 69 tissue samples from frozen or ethanol-preserved material (muscle tissue; 32 birds, 37 mammals) was obtained from dead specimens collected in the framework of the Austrian Barcode of Life (ABOL) project (www.abol.ac.at; [27]), from museum collections (Natural History Museum Vienna, Biologiezentrum Linz), or meat (cattle, wild boar) bought at a supermarket (Interspar, Wienerstrasse, Graz, Austria). These 69 samples included representatives of all Central European mammal orders and families, and all but one (Coraciiformes) bird orders breeding in Central Europe. Bird orders that only include winter guests or rare vagrants to Central Europe–Gaviiformes, Phoenicopteriformes, Procellariiformes, Pteroclidiformes–are not represented in our taxon sample (Table 2). To further test the performance of our new primer sets with dry historical material, we additionally sampled tanned hides of 14 mammal species and stuffed hides of 13 mammal and 6 bird species at the NHM Vienna (Table 3). If possible, toe pad or wing (in the case of bats) tissue was used.

**Table 2:** Fresh, frozen or ethanol preserved samples of birds (representatives of 18 orders) and mammals (representatives of 6 orders) used to test the performance of the new primer sets, with information on voucher ID, fragments of the COI barcoding region that could be successfully amplified (colors refer to the fragments in Fig 1), and GenBank accession numbers.

		Segments of the COI barcoding region										
Species	Voucher ID^1^	Start	In1	In2		In3		In4		End		GenBank Acc. No.
**Birds**												
*Apus apus* (Apo)	NMW 95220									A1		KY754482
*Aquila heliaca* (Acc)	NMW Tsk8806									A1		KY754483
*Ardea cinerea* (Pel)	NMW 95269									A1		KY754483
*Bubo bubo* (Str)	NMW 96993									A1		KY754483
*Buteo buteo* (Acc)	NMW Sk11361									A1		KY754483
*Caprimulgus europaeus* (Cap)	NMW Sk9700									A1		KY754491
*Carduelis carduelis* (Pas)	ENR663									A1		KY754492
*Chroicocephalus ridibundus* (Cha)	G 1110									A1		KY661875
*Ciconia ciconia* (Cic)	ENR658									A2		KY754495
*Columba palumbus* (Col)	ENR666									A2		KY754497
*Corvus monedula* (Pas)	NMW 97965									A1		KY754498
*Cuculus canorus* (Cuc)	NMW Sk8428									A1		KY754498
*Cygnus olor* (Ans)	ENR655									A1		KY754502
*Dendrocopos major* (Pic)	ENR660									A1		KY754503
*Erithacus rubecula* (Pas)	ENR667									A1		KY754507
*Falco tinnunculus* (Fal)	ENR659									A1		KY754508
*Hirundo rustica* (Pas)	ENR651									A1		KY754510
*Jynx torquilla* (Pic)	G 518									A1		KY661876
*Motacilla alba* (Pas)	NMW Sk11330									A1		KY754516
*Otis tarda* (Oti)	NMW Tsk8715									A1		KY754526
*Perdix perdix* (Gal)	NMW 96059									A1		KY754528
*Phalacrocorax carbo* (Pel)	G 624									A1		KY661877
*Podiceps cristatus* (Pod)	NMW Sk9712									M3		KY754538
*Prunella modularis* (Pas)	ENR665									A1		KY754541
*Rallus aquaticus* (Gru)	G 849									A1		KY661878
*Scolopax rusticola* (Cha)	NMW Sk10060									A1		KY754548
*Streptopelia decaocto* (Col)	NMW 95649									A1		KY754551
*Strix aluco* (Str)	NMW 11352									A1		KY754552
*Tadorna tadorna* (Ans)	ENR649									A1		KY754553
*Turdus merula* (Pas)	ENR653									A1		KY754556
*Upupa epops* (Buc)	G 661									A1		KY661879
*Vanellus vanellus* (Cha)	NMW Sk11151									A1		KY754558
**Mammals**												
*Apodemus flavicollis* (Ro)	NMW 69080									M1		KY754481
*Arvicola amphibius* (Ro)	NMW 68442									M3		KY754485
*Bos taurus* breed (Ce)	-									M4		KY661880
*Capreolus capreolus* (Ce)	NMW F 2732		x							A2	M4	KY754489
*Capreolus capreolus* (Ce)	SLJG 40784									M4		KY754490
*Castor fiber* (Ro)	NMW 68188									M1		KY754493
*Cricetus cricetus* (Ro)	NMW 68512									M1		KY754499
*Crocidura suaveolens* (Eu)	NMW 68240									M4		KY754500
*Erinaceus roumanicus* (Eu)	NMW 69082									A2	M1	KY754506
*Felis catus* (Ca)	-									M4		KY661882
*Glis glis* (Ro)	NMW 69079									M1		KY754509
*Lepus europaeus* (La)	NMW 69075									M4		KY754511
*Lutra lutra* (Ca)	NMW 68226									M1		KY754512
*Martes foina* (Ca)	NMW 68990							x^2^		M1		KY754515
*Martes foina* (Ca)	NMW 68228							^2^		M1		KY754514
*Muscardinus avellanarius* (Ro)	NMW 69081									M1		KY754517
*Mustela putorius* (Ca)	NMW F 2735									M1		KY754518
*Myotis mystacinus* (Ch)	NMW 68355									M3		KY754520
*Myotis nattereri* (Ch)	OLML 2005/118			x				X		M3		KY754521
*Neomys anomalus* (Eu)	OLML 2013/183									M3		KY754522
*Nyctalus noctula* (Ch)	NMW 68359			x						M3		KY754523
*Nyctalus noctula* (Ch)	OLML 2005/100									M3		KY754524
*Ondatra zibethicus* (Ro)	NMW 68327									M3		KY754525
*Pipistrellus kuhlii* (Ch)	NMW 68346			x						M3		KY754530
*Pipistrellus nathusii* (Ch)	NMW 68348		x	x		x				M3		KY754531
*Pipistrellus pipistrellus* (Ch)	NMW 68447			x						M4		KY754532
*Pipistrellus pygmaeus* (Ch)	NMW 68352			x						M4		KY754533
*Pipistrellus pygmaeus* (Ch)	NMW 69078			x						A2	M4	KY754534
*Plecotus auritus* (Ch)	OLML 2005/514			x						x		KY754535
*Plecotus auritus* (Ch)	OLML 2009/447									M4		KY754537
*Procyon lotor* (Ca)	NMW 68071									A2	M1	KY754539
*Rattus norvegicus* (Ro)	NMW 68438									M1		KY754542
*Rupicapra rupicapra* (Ce)	NMW 68065									M1		KY754546
*Sciurus vulgaris* (Ro)	NMW 69074									M1	M4	KY754547
*Spermophilus citellus* (Ro)	NMW 68222									M4		KY754550
*Sus scrofa* (Ce)	-									A1		KY661881
*Talpa europaea* (Eu)	NMW 68331									M3		KY754555
*Vespertilio murinus* (Ch)	NMW 68353									M3		KY754559

Abbrevations in parentheses following the species names refer to higher bird or mammal taxa. Birds: Apo, Apodiformes; Acc, Accipitriformes; Ans, Anseriformes; Buc, Bucerotiformes; Cap, Caprimulgiformes; Cha, Charadriiformes; Cic, Ciconiiformes; Col, Columbiformes; Cuc, Cuculiformes; Fal, Falconidae; Gal, Galliformes; Gru, Gruiformes; Oti, Otidiformes; Pas, Passeriformes; Pel, Pelecaniformes; Pic, Piciformes; Pod, Podicipediformes; Str, Strigiformes; Mammals: Ca, Carnivora; Ce, Cetartiodactyla; Ch, Chiroptera; Eu, Eulipotyphla; La, Lagomorpha; Ro, Rodentia.

x, fragment could not be amplified.

For the end-fragment, the exact primer combination used is given (see Table 1).

^1^, codes indicate the collections where the voucher specimen is deposited: NMW & ENR, Natural History Museum Vienna; OLML, Oberösterreichisches Landesmusseum Linz; SLJG, Steiermärkisches Landesmuseum Joanneum Graz; G, DNA sample obtained from the Natural History Museum Vienna, but no voucher specimen is available.

^2^, only nuclear mitochondrial pseudogene (numt) was amplified with standard primer pair M_int4.

**Table 3:** Stuffed and tanned hides tested with the new primer set, with information on voucher ID, preservation method, collection year, fragments of the COI barcoding region that could be successfully amplified (colors refer to the fragments in Fig 1) and GenBank accession number.

				Segments of the COI barcoding region										
Species	Voucher ID*	Preservation	Coll. year	Start	Int1	Int2		Int3		Int4		End		GenBank Acc. No.
**Birds**														
*Bombycilla garrulus* (Pas)	NMW 73115	S	1975	x	x	x		x		x		x		
*Chroicocephalus ridibundus* (Cha)	NMW 78277	S	1983									A1		KY754494
*Cinclus cinclus* (Pas)	NMW 94594	S	2004									A1		KY754496
*Perdix perdix* (Gal)	NMW 78412	S	1981									A1		KY754527
*Picus viridis* (Pic)	NMW 77062	S	1981					x				A1		KY754529
*Tyto alba* (Str)	NMW 82773	S	1983			x		x				A2		KY754557
**Mammals**														
*Barbastella barbastellus* (Ch)	NMW 42534	S	1956	x				x		x		x		KY754486
*Eptesicus serotinus* (Ch)	NMW 29403	S	1977	x		x		x				x		KY754505
*Eptesicus serotinus* (Ch)	NMW 57235	S	1997	x								M4		KY754504
*Martes foina* (Ca)	NMW 68272	S	2013							^1^		M3		KY754513
*Mustela putorius* (Ca)	NMW 68263	S	2012									M3		KY754519
*Ondatra zibethicus* (Ro)	NMW 68327	S	2008									M3		KY754525
*Plecotus auritus* (Ch)	NMW 30392	S	1980	x	x	x						x		KY754536
*Rattus rattus* (Ro)	NMW 54331	S	1990									M3		KY754543
*Rhinolophus ferrumequinum* (Ch)	NMW 52173	S	1993	x	x	x						x		KY754545
*Rhinolophus ferrumequinum* (Ch)	NMW 54795	S	1954	x		x		x				x		KY754544
*Sicista betulina* (Ro)	NMW 65926	S	2004									A2	M3	KY754549
*Talpa europaea* (Eu)	NMW 66719	S	2007					x				A2	M3	KY754554
*Vulpes vulpes* (Ca)	NMW 62232	S	1999									A2		KY754560
*Canis lupus* (Ca)	NMW 52409	T	1990	x	x	x		x		x		x		
*Capra ibex* (Ce)	NMW 42951	T	1976	x	x	x		x		x		x		
*Castor fiber* (Ro)	NMW 68175	T	2010	x	x	x		x		x		x		
*Cervus elaphus* (Ce)	NMW 306	T	1930	x	x	x		x		x		x		
*Lutra lutra* (Ca)	NMW 68162	T	2012	x	x	x		x		x		x		
*Marmota marmota* (Ro)	NMW 64566	T	2002	x	x	x		x		x		x		
*Meles meles* (Ca)	NMW 41073	T	1988	x	x	x		x		x		x		
*Myocastor coypus* (Ro)	NMW 8041	T	?	x	x	x		x		x		x		
*Procyon lotor* (Ca)	NMW 66311	T	2006					x				A2	M3	KY754540
*Rupicapra rupicapra* (Ce)	NMW B5026	T	1937	x	x	x		x		x		x		
*Rupicapra rupicapra* (Ce)	NMW 64127	T	1980–1989	x	x	x		x		x		x		
*Sus scrofa* (Ce)	NMW B4145/3203	T	1932	x	x	x		x		x		x		
*Ursus arctos* (Ca)	NMW 67301	T	2009	x	x	x		x		x		x		
*Vulpes vulpes* (Ca)	NMW 62231	T	1999	x	x	x		x		x		x		

Abbrevations in parentheses following the species names refer to bird or mammal orders: Birds: Cha, Charadriiformes; Gal, Galliformes; Pas, Passeriformes; Pic, Piciformes; Str, Strigiformes; Mammals: Ca, Carnivora; Ce, Cetartiodactyla; Ch, Chiroptera; Eu, Eulipotyphla; Ro, Rodentia.

*, all voucher specimens are deposited at the Natural History Museum Vienna.

Black color, fragments amplified using A2f2 and In3r; x; fragment could not be amplified.

For the end-fragment, the exact primer combination used is given (see Table 1).

S, stuffed hide; T, tanned hide.

^1^, only nuclear mitochondrial pseudogene (numt) was amplified with standard primer pair M_int4.

### DNA extraction

Total genomic DNA of frozen or ethanol-preserved material was extracted by means of a rapid Chelex protocol [28]. DNA extraction from samples obtained from skins and stuffed hides was done using the DNeasy Blood and Tissue Kit (Qiagen, Hilden, Germany) and following the protocol of [29] with two slight modifications: i) as a first step, skin samples were rehydrated in TE buffer for 24 hours at room temperature; and ii) prior to digestion, rehydrated samples underwent several washing steps (similar to [30])—twice in sterile HPLC water, then twice in absolute ethanol and again two times in HPLC water. Finally, samples were rinsed with HPLC water before they were minced into fine pieces. Final elution was conducted in 100 μl AE buffer. All plastic material used for extraction of museum samples was exposed to UV light prior to its use and all pre-PCR steps were performed in a separate ‘clean’ room with positive air pressure dedicated to working with low-quality samples. Negative controls were always included.

### PCR amplification and DNA sequencing

Amplification via polymerase chain reaction was conducted in 25 μl PCR reactions containing 1X PCR Buffer, 1.5 mM MgCl2, 0.2 mM dNTPs, 0.2 μM primer, 2 U Platinum^®^ Taq DNA Polymerase (Invitrogen) and 1–2 μl or 5 μl of DNA extract of fresh (Chelex extraction) or museum (Kit extraction) samples, respectively. PCR conditions followed the manufacturer’s protocol for the polymerase used. We employed a two-step PCR protocol with an initial denaturation step at 94°C for 2 minutes, followed by 8 cycles at 94°C for 30 seconds, 46 or 48°C for 30 seconds and 1 minute at 72°C, plus another 27 cycles at 94°C for 30 seconds, 48 or 50°C for 30 seconds and 1 minute at 72°C (annealing temperatures used differed among the different primer combinations; see Table 1). A final extension step was included at 72°C for 7 minutes. To verify the success of the PCR, amplification products were electrophoresed on a 2% agarose gel. Successfully amplified products were purified using the commercial PCR cleanup kit ExoSAP-IT^®^ (Affymetrix) following the manufacturer's instructions. Purified PCR products were sequenced bidirectionally in 10 μl sequencing reactions using the BigDye^®^ Sequence Terminator v3.1 Cycle Sequencing Kit (Applied Biosystems), applying the protocol described in [31]. Sequencing products were purified with Sephadex™ G-50 (Amersham Biosciences) using the manufacturer´s standard protocol, and visualized on an ABI 3130xl capillary sequencer (Applied Biosystems). If amplifications failed, PCRs were repeated twice, prior to testing alternative primer combinations. To test, whether it is indeed necessary to amplify and sequence the barcoding region of the museum samples in short overlapping fragments, we also tried to amplify the entire barcoding region in 30 of the museum samples.

DNA sequences were aligned in MEGA 6.06 [32]. Ambiguous sites were corrected manually and final fragments were verified through BLAST search in GenBank. Sequences are available from GenBank under the accession numbers listed in Tables 2 & 3. Furthermore, for all DNA barcode compliant samples, detailed specimen data records and sequence information (including trace files) were uploaded to BOLD (www.boldsystems.org) and are publicly available in the project titled ‘ABOL—museum primers (birds & mammals), MPBM’.

## Results

We designed and tested sets of 5’-M13-tagged PCR primers that reliably amplify the DNA-barcoding region in short (108–183 bp) overlapping DNA fragments in Central European bird and mammals orders (Fig 1, Table 2). The primer sets were first tested on a range of modern samples with representatives of 18 bird and all 5 mammal orders occurring in Central Europe. The universal primer sets for the starting and first internal region worked well for all samples except for one bat species (univ_int1 in Nathusius’ pipistrelle, *Pipistrellus nathusii*). Primer pair A_int2 amplified the majority of fresh bird samples as well as all fresh mammal samples, except for cattle (*Bos taurus*), black rat (*Rattus rattus*) and the majority of bats. Samples that did not amplify with this primer pair worked well with either primer pair A_int2_2 or M_int2 (Table 2), again with the exception of bats. The universal primer pair for the third internal region worked well in all samples, again with the exception of Nathusius’ pipistrelle. For the same region, our newly designed primer pair M_int3 worked well for most mammals (except bats). Primer pair M_int4 amplified the fourth internal region in most mammals. With the exception of Natterer’s bat (*Myotis nattereri*) those few samples that did not work with this primer pair could be amplified with A_int4, which worked well in all birds. In birds, the last fragment was amplified either with primer pair end_A1 or end_A2, or in the case of the great crested grebe (*Podiceps cristatus*) with end_M3. In mammals, this region proved to be more difficult to amplify, and various primer pairs were employed to amplify it (again with the exception of some bat species).

The primer sets were also tested on 19 stuffed hide (13 mammal & 6 bird species) and 14 tanned hide (14 mammal species) samples. The age of these specimens ranged from 2 to 61 years (Table 3; sampling and DNA extraction year in 2015). With the exception of bats, for which hardly any PCR products were obtained, and the waxwing (*Bombycilla garrulus*), for which no fragments could be amplified, probably due to insufficient DNA amount or quality, amplification and sequencing success was high in the stuffed hide samples, with only single fragments lacking in European green woodpecker (*Picus viridis*) and European mole (*Talpa europaea*). Two regions failed to amplify in the barn owl (*Tyto alba*). Contrary to the high amplification and sequencing success of stuffed hide samples, none of the tanned hide samples worked, with the exception of the raccoon (*Procyon lotor*), for which all except the fourth fragment were successfully amplified and sequenced. As all fragments were amplified in fresh/ethanol preserved European mole and raccoon samples and representatives of the Piciformes and Strigiformes (Table 2), we attribute the unsuccessful amplification of single fragments in the aforementioned samples to DNA damage rather than issues with primer binding. In contrast, the poor amplification success in bats in general might be due to poor primer binding and/or very low amounts of template DNA. For none of the 30 museum samples (stuffed or tanned hides) tried we were able to amplify the entire barcoding region in one piece, whereas the one ethanol-preserved sample used as a positive control (NMW 69075) worked well (S1 Fig).

For some species, two or three samples were barcoded. The maximum number of substitutions observed within a species was three in the roe deer (*Capreolus caproeolus*; DNA extracted from ethanol preserved tissue; two samples), whereas zero or one substitution was observed in most other species. For the muskrat (*Ondatra zibethicus*) specimen NMW 68327, we obtained barcodes from both ethanol preserved tissue and the stuffed hide, and both sequences were identical.

With the exception of the fourth internal region in stone martens (*Martes foina*), amplified with primer pair M_int4, no internal stop codons were observed, and BLAST searches in GenBank resulted in clear matches with published COI sequences, confirming that we have not sequenced mitochondrial pseudogene copies integrated into the nuclear genome which appear to be quite common in mammals and birds [33,34]. Employing the alternative primer pair A_int4 in stone martens produced the correct sequence for this fragment (Table 2, S1 Data).

## Discussion

**Fig 2:**
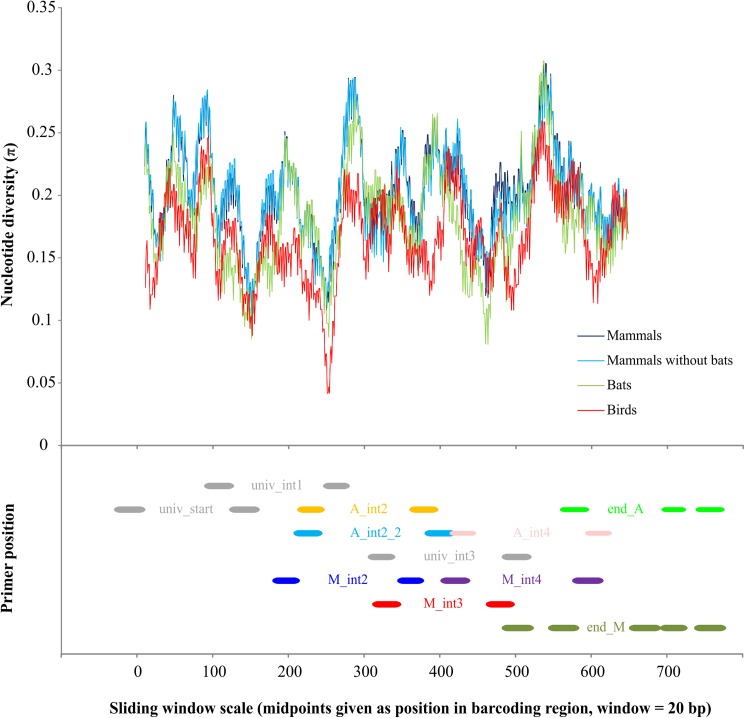
Sliding window analysis of the alignment used for designing primers (S1 Table), showing levels of nucleotide diversity along the barcoding region, with the position of primer binding regions shown in the lower panel.

The high level of variability in the COI barcoding region makes it difficult to design internal PCR primers for amplifying the entire barcoding region in short overlapping fragments, a necessity for barcoding old/degraded DNA samples typical of for example historical museum material. Designing internal COI primers that work across a wide range of taxa is a particular challenge. In this study we provide a new primer set that amplifies the barcoding region of Central European birds and mammals in six overlapping fragments. Our new primer set seems to work well in all Central European bird and mammal orders, with the notable exception of bats, for which hardly any full barcodes could be obtained. Why the barcoding success of bats was so low as compared to other mammal orders is still unclear, but some regions in the COI of bats are much more variable than in other mammalian taxa. This is especially true for the binding region of primers M2f and A2f1 (Fig 2), potentially explaining the extremely low amplification success for fragment M_int2 and A_int2 in bats. In addition to the universal starting region, two of the newly designed primer pairs, those amplifying fragments int1 and int3, appear to be universal for Central European birds and mammals (with the exception of some bats). Whether these fragments are suitable as mini-barcodes for metabarcoding approaches [35–37] remains to be tested.

Our new primer set worked well with historical museum material, in particular DNA samples obtained from stuffed hides. In general amplification success was higher in more recent samples. However, sample age does not appear to be the sole determiner of DNA quality [11] as there is no linear relationship between age and fragmentation (reviewed in [38]). Instead it is supposed that preservation methods, storage conditions or desiccation rate are more likely responsible for DNA quality and amplification success of museum material ([38] and citations therein). The crucial role of preservation methods becomes evident also from our results. Whereas, in general, samples obtained from stuffed hides worked well, tanned hide samples failed to amplify in all but one case. Reagents used in the tanning process, for example chromium(III) sulfate, potassium alum, vegetable tannins, salt or aldehydes can cause DNA degradation and, beyond that, can have negative effects on enzymatic reactions required during DNA isolation and amplification [39,40]. Inquiries as to which tanning agents were used at the Natural History Museum Vienna revealed that until the second half of the 20th century, tanned hide samples were commonly preserved with potassium alum. After this, the museum generally applied chromium(III) sulfate, a widely used tanning agent, as preservative. On the contrary, stuffed bird and mammal individuals are preserved as dry study skins, with the flayed skin treated with absorbents and then filled with any kind of material (e.g. cotton, plant fibers etc.). Thus, this difference in the preservation method used appears to be the most likely cause for the observed differences in amplification success between tanned and stuffed hides.

We added 5’-M13-tags to our PCR primers. The addition of such 5’-tags provides clear advantages over the use of non-tagged primers, as these 5’-tags allow for time- and cost-efficient large–scale sequencing. As only two primers–forward and reverse tags—are required for sequencing instead of the large number of individual PCR primers, this greatly reduces the costs and preparation time of sequencing reactions, thus facilitating high-throughput Sanger sequencing of the many short fragments amplified in the process of DNA barcoding of historical museum samples.

Recently, significant advances have been made in the use of next generation sequencing (NGS) approaches for barcoding both fresh and historical material, significantly reducing time and costs for conducting DNA-barcoding studies [41–43]. Thus far, one of the major impediments to using NGS for DNA-barcoding activities has been the typically short read lengths generated by these approaches that did not permit the sequencing of full DNA barcodes, such that so-called mini-barcodes have been typically used for specimen identification or metabarcoding applications employing NGS approaches (e.g., [24,44–47]). However, recently, increased read lengths and the development of protocols for efficiently sequencing samples in several short overlapping fragments has made NGS a realistic and cost-effective alternative to Sanger sequencing for DNA barcoding [41–43]. As NGS barcoding approaches require much less template DNA than Sanger-sequencing-based DNA-barcoding [43,48], NGS seems particularly promising for barcoding historical museum material. Indeed, large numbers of historical samples, including type material, of various arthropod orders have already been successfully barcoded using NGS [42,43]. Considering these recent developments, employing our new primer sets with already existing NGS protocols [43] opens a possibility of efficiently barcoding birds and mammals from historical museum collections. In some of our historical samples, single fragments failed to amplify, most likely not because of poor primer binding but because of low template concentration, a problem that might be possibly overcome by using NGS.

## Conclusions

Barcoding historical bird and mammal specimens has long been a tedious and time-consuming task, prohibiting their use in large-scale barcoding projects. This study established a new primer set for amplifying the DNA-barcoding region in Central European birds and mammals in six short overlapping fragments, which allows for the efficient barcoding of the many treasures present in museum collections, such that barcoding activities need not rely solely on fresh, frozen or ethanol-preserved material. Applying our new primer set in recently established NGS protocols promises to further increase the efficiency of barcoding old bird and mammal specimens. Nonetheless, unless a project requires the inclusion of type material—which would be beneficial for barcoding projects in any case–or taxa for which no fresh material is available, we advocate the use of fresh material whenever possible.

## Supporting information

S1 Data Sequences of mitochondrial pseudogene copies integrated into the nuclear genome of stone martens (*Martes foina*), obtained with primer pair M_int4, and correct stone marten barcode fragments obtained with the alternative primer pair A_int4. (FAS)

S1 Fig Agarose gel picture showing the lack of amplification success of the entire barcoding region in stuffed and tanned hide samples. The only sample that worked was the positive control, an ethanol-preserved European hare sample (NMW 69075). For more information on samples (species, preservation method) see Table 3.
(TIF)

S1 Table Sequences used for designing primers. (XLSX)
