# Supplementary figures and images for: Opening the treasure chest: A DNA-barcoding primer set for most higher taxa of Central European birds and mammals from museum collections
Source: PLoS One. 2017 Mar 30;12(3):e0174449. doi: 10.1371/journal.pone.0174449 (PMC5373577; doi:10.1371/journal.pone.0174449)

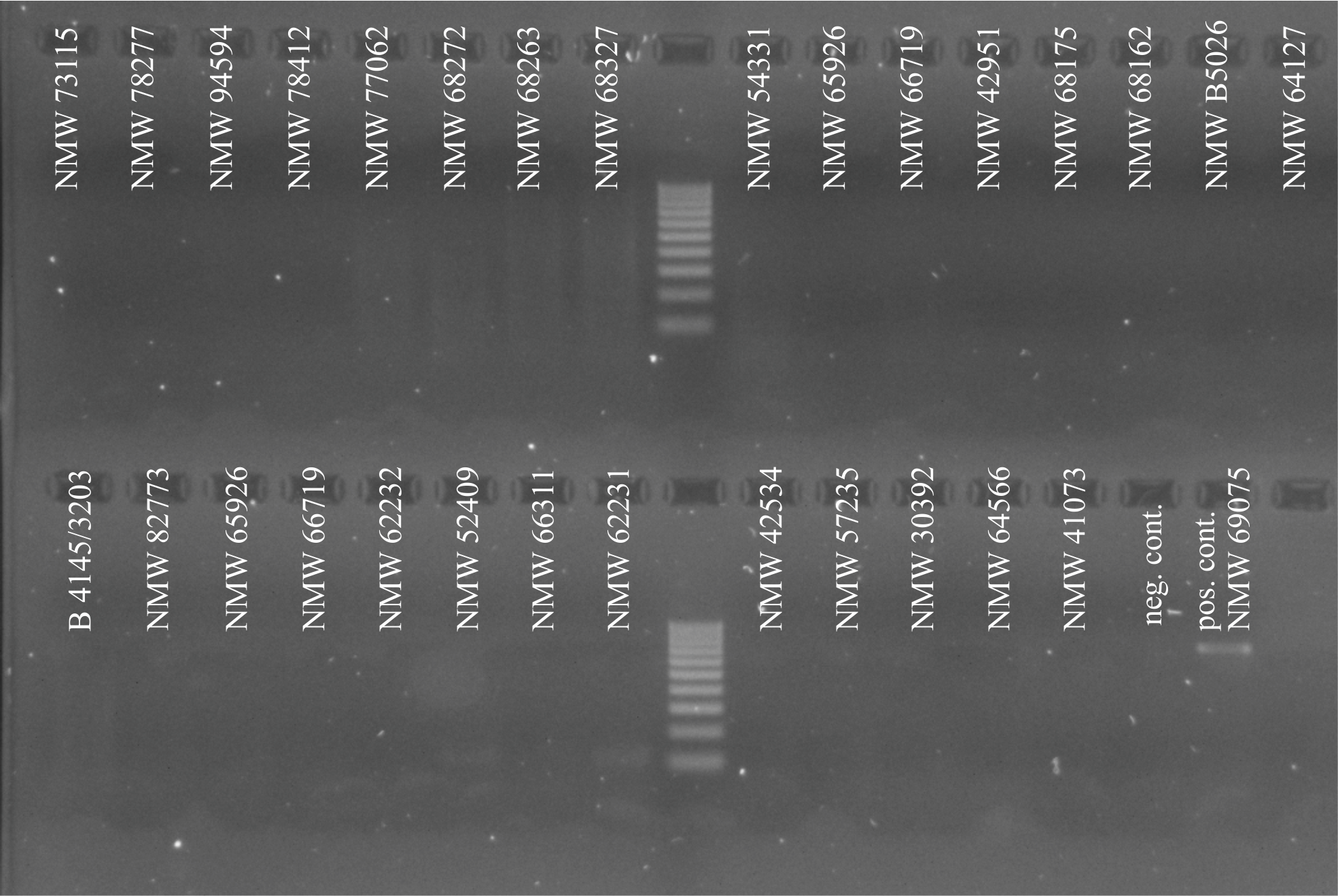

Supplement: S1 Fig — The only sample that worked was the positive control, an ethanol-preserved European hare sample (NMW 69075). For more information on samples (species, preservation method) see Table 3. (TIF) [file pone.0174449.s002.tif]
